# Harnessing genetically engineered cell membrane-derived vesicles as biotherapeutics

**DOI:** 10.20517/evcna.2023.58

**Published:** 2024-01-31

**Authors:** Xiaohong Li, Yuting Wei, Zhirang Zhang, Xudong Zhang

**Affiliations:** ^1^Shenzhen Key Laboratory for Systems Medicine in Inflammatory Diseases, School of Medicine, Sun Yat-Sen University, Shenzhen 518107, Guangdong, China.; ^2^Department of Pharmacology, Molecular Cancer Research Center, School of Medicine, Sun Yat-sen University, Shenzhen 518107, Guangdong, China.

**Keywords:** EVs, genetically engineering, biomedicine, drug delivery

## Abstract

Cell membrane-derived vesicles (CMVs) are particles generated from living cells, including extracellular vesicles (EVs) and artificial extracellular vesicles (aEVs) prepared from cell membranes. CMVs possess considerable potential in drug delivery, regenerative medicine, immunomodulation, disease diagnosis, *etc*. owing to their stable lipid bilayer structure, favorable biocompatibility, and low toxicity. Although the majority of CMVs inherit certain attributes from the original cells, it is still difficult to execute distinct therapeutic functions, such as organ targeting, signal regulation, and exogenous biotherapeutic supplementation. Hence, engineering CMVs by genetic engineering, chemical modification, and hybridization is a promising way to endow CMVs with specific functions and open up novel vistas for applications. In particular, there is a growing interest in genetically engineered CMVs harnessed to exhibit biotherapeutics. Herein, we outline the preparation strategies and their characteristics for purifying CMVs. Additionally, we review the advances of genetically engineered CMVs utilized to target organs, regulate signal transduction, and deliver biomacromolecules and chemical drugs. Furthermore, we also summarize the emerging therapeutic applications of genetically engineered CMVs in addressing tumors, diabetes, systemic lupus erythematosus, and cardiovascular diseases.

## INTRODUCTION

CMVs are membrane particles secreted or prepared from almost all living cells, including prokaryotes and eukaryotes, which are delimited by the phospholipid bilayer membrane-bound structure^[1]^. Classified by source, CMVs could be divided into naturally occurring EVs and aEVs prepared from cell membranes. Given differences in derivation and biomolecular components, the EVs are classified into three main subtypes (exosomes, ectosomes, and apoptotic EVs). Exosomes are the most-studied type of EVs, and they are secreted from multivesicular bodies (MVBs) *via* the endosomal pathway, while ectosomes are outward shedding from the surface of cell membranes directly^[2,3]^. Apoptotic EVs are released as fragments of cells undergoing apoptosis^[4]^. EVs spontaneously sort biomacromolecule cargos such as protein and nucleic acids into nanovesicles and deliver them to recipient cells without degradation and biological barriers, interpreted as information transmitters in normal physiological function, immune response, and disease development^[1,3,4]^_._ Although the mechanisms of cargo sorting into EVs are still not fully understood, there are several instances in which EVs are utilized to load endogenous biomacromolecules including protein, DNA, and RNA^[5]^. The aEVs are generally obtained through manual methods such as ultrasonic extrusion *in vitro*, which also maintain the profile of EVs. In contrast to polymer-based nanoparticles, CMVs possess properties including stable lipid bilayer structure, favorable biocompatibility, and lower toxicity, which endow CMVs with great potential as drug carriers utilized for therapeutic purposes^[5,6]^.

Nevertheless, natural CMVs have inherent limitations that have restrained scientists’ research and restricted the application of CMVs as biotherapeutics. For instance, the most significant constraint is yield and heterogeneity^[7]^. The heterogeneity of CMVs caused by different original cells, discrepant biogenetic routes, and distinction in purification strategies, posed challenges to deeply understanding the components and functional characteristics of the distinct secreted components, confounded our analyses and limited their efficiency as biotherapeutics^[7,8]^. Moreover, CMVs innately have biological components derived from original cells and could deliver information to recipient cells through the progress of intercellular communication^[9]^. Although most of CMVs inherit certain attributes of the original cells, it is still difficult for them to execute distinct therapeutic functions, such as inadequate organ targeting, insufficient signal regulation, and a lack of exogenous biological therapeutic supplementation.

Further studies have demonstrated that engineered CMVs are promising nanomedicine with a broad range of specific applications^[10,11]^. Through strategies including genetic engineering, click chemistry, and other engineering methods such as hybridization, sonication, and electroporation^[5]^, CMVs’ internal and membrane surface components are optimized, thus enabling enhanced or brand-new functions. Hence, genetically engineered CMVs harnessed to exhibit biotherapeutics elicit increasing interest. Herein, we outline the preparation strategies and their characteristics for purifying CMVs. Additionally, we review the advances of genetically engineered CMVs utilized to target organs, regulate signal transduction, and deliver biomacromolecules and chemical drugs. Furthermore, we also summarize the emerging therapeutics involving genetically engineered CMVs in the treatment of tumors, diabetes, systemic lupus erythematosus, and cardiovascular diseases, which will be beneficial to a better understanding of the development and challenges of this field.

## PREPARATION OF CELL MEMBRANE VESICLES

### Purification methods of EVs

**Figure 1:**
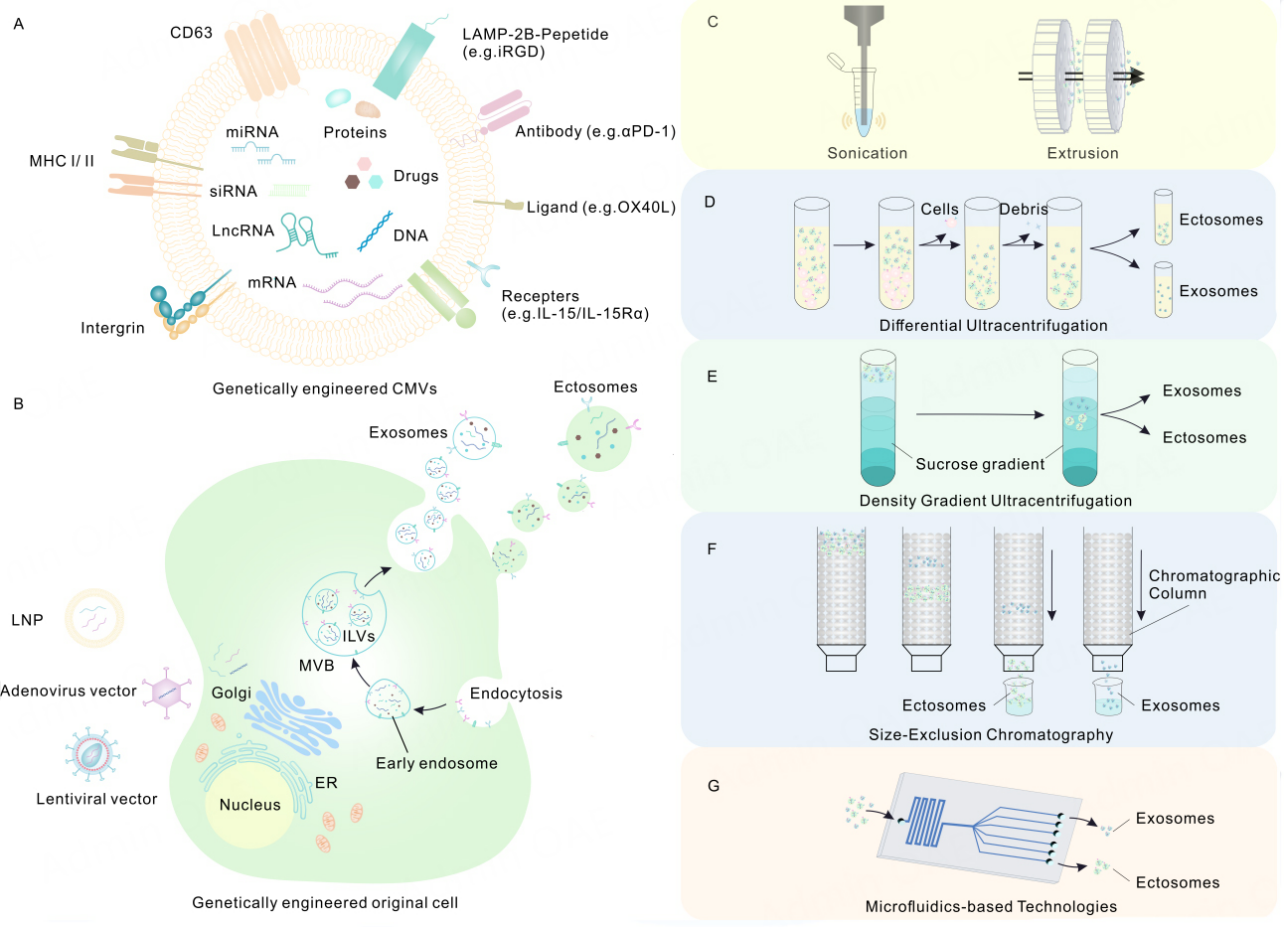
Schematic diagram of genetically engineered modification mechanism of CMVs and series of strategies for separation and purification. (A) Strategies for modifying CMVs by genetic engineering; (B) Transfection and delivery of gene or microRNAs sequence via vectors including LNPs and vectors are core steps in the process of cell genetic engineering. Hereafter, the donor cell’s biosynthetic machinery is employed to produce specific biomacromolecules which will execute specific functions inside and outside the cell. Thus, CMVs generated from original cells were modified to load exogenous biotherapeutics and used for disease treatment through genetic engineering methods; (C) The aEVs are acquired by series of sonication and extrusion; (D-E) Ultracentrifugation depends on size or molecular weight employing a series of centrifugal forces and duration, including two types of preparative ultracentrifugation--differential ultracentrifugation and density gradient ultracentrifugation; (F) Size-Exclusion Chromatography; (G) Microﬂuidics-based Technologies. LNP: lipid nano particle; ILVs: intraluminal vesicles; MVBs: multivesicular body; ER: endoplasmic reticulum; CMVs: cell membrane-derived vesicles.

EVs could be generated *via* normal biogenesis and released to the cellular space. We have developed a better understanding of mechanisms during this process. Exosomes originate from the formation of endosomes, assumed to be a heterogeneous subgroup that are secreted *via* inward membrane budding of early endosomes to generate intraluminal vesicles (ILVs), the precursor of exosomes, within MVB. Original cells released ILVs to the peripheral cytoplasm space as exosomes with a scale of 30 ~ 150 nm through power-driven membrane fusion between MVB and the plasma membrane^[4,7,12]^. Ectosomes are directly derived through outward budding from the plasma membrane. There is more than one type of ectosome with size of 150~1000 nm^[4]^. Apoptotic EVs are identified as fragments released by original cells that are undergoing apoptosis, arising from the outward blebbing of the membrane, with a size range of 100 nm ~ 5 μm^[13,14]^. Given the existence of different subpopulations of EVs with similar morphology and overlapped size ranges, the proper isolation and purification strategies are crucial for understanding EVs’ action mechanisms and are conducive to their extended application in biomedicine. Purification techniques with unique features have been adopted to facilitate EV isolation. Given that the natural EVs’ biogenesis process occurs on the genetically engineered donor cells, the purification procedures are applicable to the genetically engineered donor cells as well [Figure 1].

For method I: **Ultracentrifugation.** Ultracentrifugation remains the most widely employed purification strategy^[15]^. It briefly relies on the different size and weight characteristics of EVs for isolation, utilizing centrifugal forces and specific duration. Ultracentrifugation encompasses techniques such as differential ultracentrifugation and density gradient ultracentrifugation^[16]^. For differential ultracentrifugation: after removing cells and debris during a series of low-speed centrifugation processes, the samples undergo ultracentrifugation at 10,000× *g* to collect ectosomes and at 100,000× *g* to obtain exosomes. For density gradient ultracentrifugation: EVs are separated *via* continuous or discontinuous gradients. This method has been extensively used to purify EVs. However, it has certain limitations when isolating EVs from vesicles with comparable sedimentation velocities. Compared ultracentrifugation with density gradient ultracentrifugation, Tauro *et al.* revealed that all separated products contained nanovesicles with close size and similar expression profiles of protein markers on the membrane surface, but they had different relative abundances of particular proteins^[17]^. In addition, high-speed centrifugation typically revealed heterogeneous EV aggregates^[18]^. Compared to differential ultracentrifugation, density gradient centrifugation gives the purer exosome population^[19]^.

For method II: **Size-Exclusion Chromatography**. EVs with similar sizes are isolated together through sequential elution from the specific column^[16]^. The porous gel ﬁltration polymer as the stationary phase allows differential elution in order: bigger particles tracing fewer pores are eluted ﬁrstly, and the smaller particles follow closely behind the bigger ones^[14]^. In contrast with EVs isolated by ultracentrifugation, the golden standard for EV isolation, the yielded EVs using size-exclusion chromatography possess more intact biophysical properties and bear higher functionality^[20]^. In a study conducted by Sidhom *et al.,* size-exclusion chromatography was reviewed for its arrestive purity, functionality, replicability, and scalability, without the specialized equipment or expertise^[14]^. Recently, the practice of applying size-exclusion chromatography to EV purification has remarkably increased.

For method III: **Microﬂuidics-based Technologies.** Microﬂuidics-based isolation technologies depend on the immunoaffinity, size, and density of EVs. These liquid biopsy systems employ a series of methods to isolate EVs, such as immunofluorescence, nanoporous membrane sieving, nanowires on microcolumns for exosome capture, acoustic-based microfluidic devices, and viscoelastic microfluidic sorting^[21]^. One of the advantages of using microﬂuidic devices is high throughput and efficiency. Microﬂuidics-based isolation technologies have great potential in clinical applications, although it is not a standard isolation method.

### Preparation methods of aEVs

In addition to the methods of obtaining EVs, the cell membrane could also be extracted by mechanical methods to produce aEVs. A previous review noted that the first step of extracting the original cell membrane is processing the cells into a cell lysis mixture through mechanical homogenization^[22]^.

For method I: **extrusion**. The original cells are successively lysed and extruded several times through polycarbonate membrane filters of different diameters using a hand-held extruder to harvest aEVs with a core-shell structure^[23]^. The drugs and other target biotherapeutics could be mixed with original cells in the extrusion procedure so that the cargo-loaded aEVs are collected in parallel. In an earlier study, the cell suspension was sequentially extruded three times through polycarbonate membrane filters and the drug-free or drug-loaded aEVs were acquired for carrying chemotherapeutics targeting the tumor^[23]^. Similarly, other studies also reported cases utilizing this approach to enable aEVs with the ability for drug delivery^[24,25]^. Jhan *et al.* reported the sequential extrusion of mixtures including the cell suspension and lipid through orifices of different diameters: 400 nm, 200 nm, and 100 nm^[26]^. At each step, the mixtures were squeezed more than 25 times manually. After that, the engineered extracellular vesicles were harvested to establish an efficient gene delivery system^[26]^.

For method II: **sonication**. The cell homogenized mixture was generally sonicated with the following settings: 20% power, 4 sec of pulsing followed by 2 sec of pause, repeated 6 times, and then cooled on ice for 2 min. After that, gradient centrifugation was carried out to harvest high-purity cell membranes^[27]^. Haney *et al.* used the following settings: 20% power, 6 cycles, 30 sec on/off each time, lasting for 3 min, with 2 min of cooling between each cycle to load chemotherapeutics into aEVs simultaneously^[25]^.

It is worth noting that the study by Wen *et al.* supports the previous studies with the notion that aEVs have comparable performance with EVs in physicochemical properties (size, morphology, and classical markers, *etc*.)^[27]^. Moreover, Chul’s research revealed that the yield of aEVs is 50-100 times more than EVs, while the cost is less than 10% of EVs^[23,27]^. However, extrusion and sonication have definite defects of compromising the membrane integrity. Therefore, some researchers choose to incubate the mixture at 37 °C to promote recovery of the impaired exosomal membrane structure^[24]^.

## GENETICALLY ENGINEERED STRATEGIES IN CELL MEMBRANE VESICLES

In the past decades, genetic engineering has been one of the most widely practiced engineering strategies, documented and tested to induce cells to express specific products. The emerging gene vectors for delivering nucleic acid fragments include viral and nonviral vectors^[22]^. Transfection and delivery of genes or RNA sequences are core steps in the process of cell genetic engineering. Hereafter, the original cell’s biosynthetic machinery is employed to produce specific biomacromolecules that will execute specific functions inside or outside the cell. Hence, genetic engineering is increasingly supposed to be highly efficient for engineering CMVs, including membrane modification and exogenous cargo loading according to requirements^[28]^. With genetic engineering, researchers have achieved their aim of feasibly loading certain biotherapeutics (protein, nucleic acid, chemical molecules, *etc*.) into original cells and then transferring them to secret EVs through the natural biogenesis process or aEVs via an artificial manufacturing process. In the subsequent sections, we focus on strategies for how CMVs may be modified to load exogenous biotherapeutics and used for disease treatment through genetic engineering methods [Table 1].

**Table 1:** Modification characteristics and advantages of genetic engineering strategies for CMVs

**Engineered strategies**	**Types**	**Modification technique**	**Modified form**	**Advantages**
Protein	Receptor or ligand, antibody and targeting peptide	Genetic engineering vectors	Surface labeling and loaded delivery	Natural pathways， Protective delivery， Specific Targeting
Chemical drug	Chemotherapeutic drugs and photosensitive materials	Incubation and targeted molecular modification on the surface		Targeting, Enhanced therapeutic efficient
Nucleic acids	DNA, siRNA, miRNA and mRNA	Electroporation, electrotransfection and genetic engineering vectors		Genome editing, efficient delivery, prolong circulation

CMVs: cell membrane-derived vesicles.

### Protein cargo loading into CMVs

In general, the biotherapeutic proteins including ligands, antibodies, and targeted peptides are primarily fused with proteins ( such as LAMP-2B, CD63, *etc*.) expressed on CMVs membrane surface^[9,10]^. Since CMVs are generated from cell membranes, the overexpressed proteins located on the cell membranes will be transferred onto them. Certainly, the aEVs prepared from the cell membrane will also display the membrane-located proteins including the overexpressed proteins. These bio-functional proteins are displayed on the CMV membrane so that these strategies could enhance the targeting and therapeutic effect for various diseases^[29]^.

#### Genetic engineering protein receptor or Ligand on the surface of CMVs

Wu *et al.* established a stable cell line with overexpression of the central nervous system (CNS) lesion -targeting ligand PDGFRα and collected the EVs^[30]^. Combined with the utilization of Bryostatin-1 (Bryo-1), the microglia pro-inflammatory phenotype in the CNS is dramatically altered and the clinical disease development of EAE mice is significantly ameliorated^[30]^. Tyrosine phosphatase-2 (SHP2) highly expressed EVs derived from MSCs were utilized to penetrate the blood-brain barrier, induce mitophagy in neuronal cells, and diminish their apoptosis, contributing to the treatment of Alzheimer's disease (AD)^[31]^. The oncolytic adenoviruses camouflaged by CMVs harboring the targeting ligands achieve the purpose of suppressing the intracorporal immune responses against oncolytic adenoviruses (OA) and enhance OA’s targeting capabilities to a greater extent^[32,33]^. The genetically modified genes of targeted protein ligands, including human epidermal growth factor (hEGF) and anti-HER2 Affibody, were introduced into cells to collect CMVs which presented tumor-targeting ligands on the membrane. The CMVs exhibit enhanced targeting capability *via* excellent ligand-mediated affinity to epidermal growth factor receptor (EGFR)or HER2 on the tumor surface^[33]^.

Single protein modification for original cells or CMVs is often of limited benefit. Thus, some reports have carried out combined modifications of multiple functional protein types for disease treatment. CMVs based on the genetically engineered dendritic cell were primarily created as a cancer nanovaccine. Dendritic cells, which were infected by recombinant adenovirus displayed the specific peptide-major histocompatibility complex class I (pMHC-I), anti-programmed death 1 (PD-1) antibody, and B7 costimulatory molecules. In this way, the CMVs distinctly improved antigen delivery and triggered a wider range of T-cell immune responses for established tumors^[34]^.

#### Overexpressing Antibody and Nanobody on CMVs

Antibody-drug conjugates (ADCs) act to target the delivery of drugs to specific tissues through high-affinity binding between antigens and antibodies, which significantly contributes to the precise delivery of biotherapeutics in many diseases. However, limited drug release efficiency and drug inactivation during drug delivery are prompting increasing concerns^[35]^. Engineered EVs may circumvent these problems because they can encapsulate the chemical drugs and protein drugs within or on the membranes of EVs. A study explores the synthetic multivalent antibodies retargeted exosomes (SMART-exosome), expressing antibodies specific for CD3 and the EGFR. The cross-linking between T lymphocytes and EGFR-positive cancer cells was promoted. Additionally, the SMART-exosome has a good performance in provoking intensive anticancer immunity *via* recruiting and activating cytotoxic T cells^[36]^. Similarly, another recent study by the same research team suggested that genetically engineered exosomes armed with not only CD3, EGFR but also the programmed death 1 and OX40 ligand (OX40L) obviously killed the EGFR-positive cancer cells and inhibited progression of established tumors^[37]^. Xue *et al.* established aEVs displaying anti-PD-1 single-chain variable fragment antibody (aPD-1-scFv) to counteract immune resistance mediated by the PD-1/PD-L1 axis in cancer^[38]^. The CPI-444 was loaded with aPD-1-scFv aEVs to simultaneously antagonize adenosine^[38]^. The CPI-444-aPD-1-scFv aEVs not only activated T cells to exhibit the capability of antitumor but also intensively increased the infiltrating T cells density, which inhibited the tumor progression and metastasis^[38]^.

#### Displaying targeting peptides on the surface of CMVs

Functional peptides have been synthesized through various peptide design technologies, which are utilized widely in the production of therapeutic agents for targeted therapy^[39]^. Surface presentation of targeting peptides on CMVs can be realized by the sequence expression of targeting peptides fused to transmembrane proteins of CMVs^[35]^. In particular, cell-specific targeting peptides can be genetically engineered at the N-terminus of LAMP-2B. The αγ integrin-specific iRGD peptide (CRGDKGPDC) was displayed on exosomes *via* fusion expression with LAMP-2B, and it enhanced the targeting efficiency of doxorubicin (DOX) to cancer cells^[40]^. Similarly, KRAS small interfering RNA (siRNA) was delivered specifically to tumors *via* fusion expression between iRGD and LAMP-2B. The results indicated that these engineered EVs led to KRAS gene expression down-regulation and tumor growth halt^[41]^. In addition, other types of fusion proteins have been reported and applied. For example, Zhu *et al.* prepared the c(RGDyK)-modified and paclitaxel (PTX)-loaded ESC-exosomes, and confirmed they have much better performance in improving the therapeutic activity of PTX *via* enhanced targeting capability *vs*. the free drug alone in glioblastoma (GBM)^[42]^. In addition, another study established a “production center” based on synovial mesenchymal stromal cells (SMSCs) that produced CRY2-ZEB1 and CIBN-CD9, two recombinant fusion proteins, which were used to generate ZEB1-loaded EVs with c(RGDfC) surface modification simultaneously for improving the bone defect regeneration^[43]^.

### CMVs loading and target delivery of chemical drug

CMVs-mediated drug delivery has the advantages of favorable biocompatibility and lower toxicity *in vivo*, which endow CMVs with great potential as carrier systems to improve drug stability in circulation and ameliorate drug accumulation in recipient sites. A study developed exosomes that target sigma receptors and are loaded with PTX, combined with an aminoethyl ethanolamine-polyethylene glycol (AA-PEG) carrier. The AA-PEG-exoPTX significantly accumulated where the tumor cells have been established and improved the drug’s therapeutic outcomes^[44]^. Ultrasonication was utilized to collect genetically engineered CMVs, sorting the photosensitizer (ICG) or DOX. The drug-loaded CMVs have a better performance on antitumor progression^[33]^. The ExoSTING is derived from EVs that were engineered to exogenously carry cyclic dinucleotide (CDN), and it was proved that it can enhance the potency of CDN and significantly activate antigen-presenting cells^[45]^.

### Engineering CMVs sequester and delivery of nucleic acid cargo

#### Transfection of exogenous DNA into CMVs

Transfection of recipient cells by plasmid vectors containing specific DNA sequences is a traditional genetic engineering method to achieve direct gene delivery, further fundamentally changing certain biological properties of recipient cells. CMVs could also be genetically modified *via* this approach. Huang *et al.* genetically modified human bone marrow-derived MSCs (HMSCs) for expressing bone morphogenetic protein 2 (BMP2), and they proved that transfected cells produced EVs that potentiated the BMP2 signaling cascade and thus enhanced osteoinductive properties^[46]^. Research indicated that the myotube formation of sarcoblast was promoted while downregulating the expression of fibrotic genes with the delivery of miR29-EVs. In tibialis anterior (TA) muscle injury model, the miR29-EVs conduced to the consolidation of primary myoblasts and host muscle^[47]^.

CMVs were also designed to enhance the CRISPR/Cas9 system for gene delivery. McAndrews *et al.* demonstrated that CRISPR/Cas9-CMVs can target the *Kras*^G12D^ and successfully suppress the proliferation and growth of tumor^[48]^. Gee *et al.* have developed an all-in-one CRISPR/Cas9 ribonucleoprotein delivery platform (NanoMEDIC) using EVs, which were efficiently involved in genome editing in various hard-to-transfect cells^[49]^. The com-com interaction occurs by forming a ternary complex consisting of CD63-Com fusion protein, com-modified sgRNA, and Cas9 or ABE, enriching Cas9 and Adenine Base Editor (ABE) RNPs into EVs. These RNP-enriched EVs exhibit high-performance genome editing capabilities and can be instantly expressed for high-efficiency and safe CRISPR genome editing^[50]^.

#### Introduction of RNA into CMVs

Recently, CMVs have emerged as a promising carrier system for transporting therapeutic nucleic acids in multiple disease models. RNA (including mRNA, miRNA, siRNA, and other non-coding RNA) is a kind of favorable cargo loaded into CMVs in order to realize certain curative effects. With the utilization of electroporation, various targeting RNA are transfected into donor cells or secreted EVs. Moreover, the aim of RNA precision delivery *in vivo* is achieved by designing a targeting system, such as that reviewed above. For example, synthesized siRNA as therapeutic is camouflaged into targeting tLyp-1 exosomes that are transfected by constructed tLyp-1-LAMP-2B plasmids in advance. After that, lung cancer or cancer stem cells uptook the gathered exosomes and synthesized siRNA working to knock down the SOX2 gene^[51]^. In another study, Bellavia *et al.* engineered HEK293T cells to display the LAMP-2B fused with Interleukin 3 (IL3) segment, and collected the IL3-LAMP-2B exosomes to load with Imatinib or with BCR-ABL siRNA, targeting CML cells and inhibiting cancer progression *in vitro* and *in vivo*^[52]^. Lu *et al.* designed a small interfering RNA against PAK4 (siPAK4) and drove them to form the nanocomplex core assembling with a photoactivatable ROS-sensitive polymer, which is further encapsulated by EVs from M1 macrophages through reaped extrusion^[53]^. The delivery of siPAK4 breached the defense of tumor-cell-intrinsic “guard” PAK4, boosted intratumoral infiltration, and elicited robust anticancer immunity^[53]^. A multifunctional biomimetic nanoplatform that combines small interfering RNA against PD-L1 (PD-L1 siRNA), Ru-TePt nanorods, and CMVs was designed. The anti-PD-L1-siRNA caused PD-L1 gene silencing and considerably actived cytotoxic T cells, which work synergistically with an enhanced reactive oxygen species provoked to trigger by Ru-TePt to trigger antitumor immune response. Hence, the Ru-TePt@siRNA-MVs nanosystems evoked the impressive immune response triggered by oxidative stress and suppressed immune resistance mediated by the immune checkpoint^[54]^. CMVs derived from MSCs encapsulated siRNAs to target the Myc and were localized to orthotopic GBM^[55]^.

## GENETICALLY ENGINEERED CELL MEMBRANE VESICLES AS DISEASE TREATMENT STRATEGY

**Figure 2:**
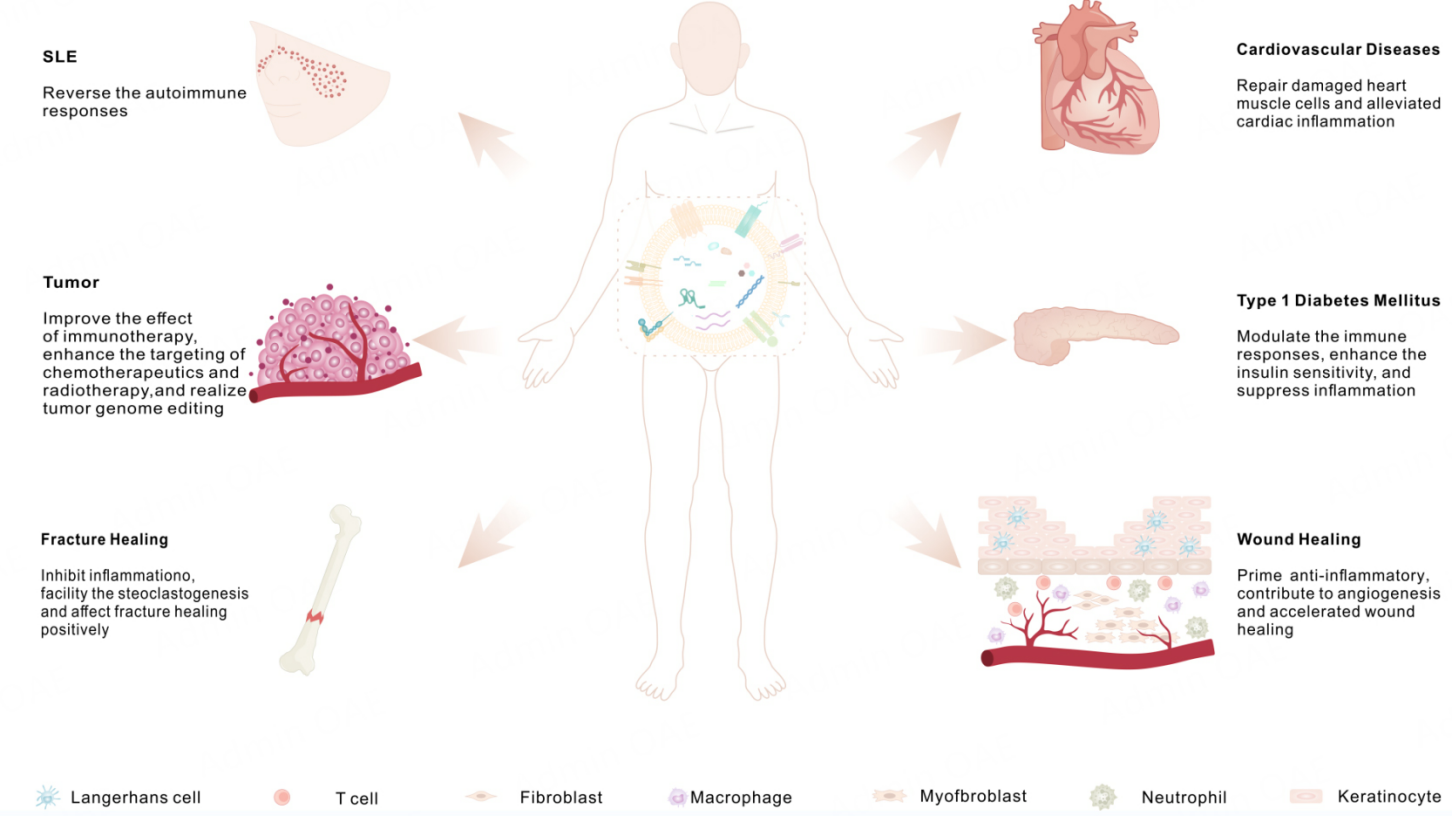
Applications in treatment of common diseases model with genetically engineered CMVs. Genetically engineered CMVs work through an overexpressed proteins or nucleic acid which function as a targeting molecules, biotherapeutics, or immune modulators in therapy for tumors, systemic lupus erythematosus, diabetes, and cardiovascular diseases. CMVs: cell membrane-derived vesicles.

Genetically engineered CMVs maintain the inherent advantages of native CMVs, including a stable lipid bilayer structure, favorable biocompatibility, and lower toxicity^[5]^. Critically, CMVs modified through genetic engineering will endow them with overexpressed protein that function as a targeting molecule, biotherapeutics, or immune modulators. Still not only such, in consideration of modification strategies’ diversity, genetically engineered cell membrane vesicles exhibit distinctive advantages in the realm of chemical drug carriers. Genetically engineered cell membrane vesicles have been inarguably meaningful for disease treatment. Herein, we focus on the advanced applications of CMVs within common disease treatment strategies [Figure 2 and Table 2].

**Table 2:** Advanced applications of genetically engineered CMVs in cancer treatment

**Disease model**	**CMVs source**	**Types**	**Engineered strategy**	**Key features**	**Mechanisms**	**Refs**
Immunotherapy	CTLL-2	EVs	PD-1	Restart the activity of CD8^+^ T cells and enhance the tumor elimination	Interrupt the PD-1/PD-L1 immune inhibitory axis	[61]
	HEK 293T	aEVs	PD-1	Reactivate CD8^+^ T cells and increase the extent of CD8^+^ in tumors	Interrupt the PD-1/PD-L1 immune inhibitory axis	[62]
	BMDC	EVs	aCD19 scFv, PD-1	Accumulate in huCD19-expressing solid tumors and reverse the immune landscape	Interrupt the PD-1/PD-L1 immune inhibitory axis	[63]
	DC2.4	aEVs	MHC I, a PD-1, B7	Eliminate established tumors	Improve antigen delivery to lymphoid organs and generate broad-spectrum T-cell responses	[34]
	Raw264.7	aEVs	aPD-1-scFv, CPI-444	Activate T cells and inhibit the tumor progression and metastasis	Interrupt the PD-1/PD-L1 immune inhibitory axis	[38]
	HEK 293T	aEVs	CD64, aPD-L1, cyclophosphamide	Inhibit the tumor growth and prolong survival time	Interrupt the immune escape mediated by PD-L1 and control the inhibition of regulatory T cells	[64]
	NIH 3T3	aEVs	IL-15/IL-15Rα, PD-1/PD-L1 inhibitor 1	Enhance the activation and proliferation of tumor-infiltrated T cells	Interrupt the PD-1/PD-L1 immune inhibitory axis	[65]
	ReN cells	EVs	siPD-L1, RGDyK	Increase CD8^+^ T cell activity, halt tumor growth and prolong survival	Enhance the targeting efficiency of RGD-EV to murine GBM, while the loaded siRNA reverse radiation-stimulated PD-L1 expression on tumor cells	[66]
Chemotherapy	imDCs	EVs	LAMP-2B, iRGD, DOX	Decelerate the tumor growth	Show excellent targeting and DOX convey capacity to αv integrin-positive cancer cells	[40]
	HEK 293T	EVs	CL4, DARS-AS1 siRNA, DOX	Demonstrate strong anti-proliferative, anti-migratory, and pro-apoptotic effects on TNBC cells.	Suppress the TGF-β/Smad3 signaling pathway-induced autophagy and increase the sensitivity of tumor cells to DOX	[73]
	Raw264.7, BMM	EVs	AA-PEG vector, PTX	Superior antineoplastic effect and increase survival times	Excellent ability to target σ receptors	[33]
	Embryonic stem cells	EVs	c(RGDyK), PTX	Inhibit the GBM growth	Improve the curative effects of PTX in GBM via enhanced targeting	[42]
Immunotherapy+ Chemotherapy	BM-MSC	EVs	galectin-9 siRNA, OXA	Reverse immunosuppressive TME, increase infiltration of antitumoral cytotoxic T lymphocytes, and increase drug accumulation in tumor site	Disrupt the galectin-9/dectin 1 axis	[74]
Photodynamic therapy + Chemotherapy	HEK293T	aEVs	hEGF or anti-HER2 Affibody, ICG, DOX	Improve antitumor therapeutic outcomes and reduce side effects	Enhance targeting capacity	[33]
Radiotherapy	MSCs	EVs	miR-34c	Inhibit tumor progression and increase the efficiency of radiotherapy		[76]
	NPC cells	EVs	miR-197-3p	Reduce the proliferation and migration of NPC cells, alleviate tumor growth, and enhance their radiosensitivity	Regulate AKT/mTOR phosphorylation activation and HSPA5-mediated autophagy	[77]
Geng therapy	HEK 293T	EVs	SOX2 siRNA	High transfection efficiency into tumor cells	Targeting tLyp-1 exosomes are successfully engineered	[51]
	HEK293T	EVs	LAMP-2B, IL-3, Imatinib or BCR-ABL siRNA	Target CML cells and inhibit *in vitro* and *in vivo* cancer cell growth	Enhance targeting capacity	[52]
	M1 macrophages	EVs	PAK4 siRNA, ROS-sensitive polymer	Robust anticancer immunity	Prime the TME through immunogenic phototherapy, boost intratumoral infiltration and immune activation	[53]

CMVs: cell membrane-derived vesicles.

### Harnessing genetically engineered CMVs in cancer treatment

#### CMVs in Immunotherapy

Immune checkpoint blockade therapy reinvigorates the host’s anti-tumor immune response for cancer treatment^[56]^. With application of genetically engineered CMVs, we have another perspective to modulate immune checkpoint therapy for cancer treatment. Cancer cells overexpress PD-L1 on the membrane, enhancing the binding ability to effector T cells with high expression of PD-1, inducing T cell exhaustion, and contributing to tumor immune escape^[57-60]^. The excellent biotherapeutics delivery capacity of genetically engineered CMVs enables therapeutic biomacromolecules to be transported to target cells with reduced immunogenicity and enhanced efficiency for immune checkpoint-based immunotherapy. A recent study reported that the constructed T cell-derived EVs displaying PD-1 on the membrane surface to interrupt the PD-1/PD-L1 immune inhibitory axis and enhance tumor elimination. The EVs effectively restarted the activity of CD8^+^ T cells. Moreover, PD-1 EVs could even kill tumor cells and directly drive tumor regression^[61]^. Similarly, the genetically engineered HEK 293T cell expressing PD-1 receptors were utilized to harvest PD-1 aEVs from an earlier research. PD-1 on the surface of aEVs not only reactivated suppressed CD8^+^ T cells but also significantly increased the extent of CD8^+^ lymphocyte infiltration in the margin of tumor by specifically binding to PD-L1 on cancer cells^[62]^. In another independent investigation, researchers genetically modified aCD19 scFv and PD-1, incorporating them onto the surface of DCs for the extraction of bi-specific EVs. The engineered EVs accumulated within huCD19-expressing tumors, and reversed the immune landscape^[63]^. Moreover, CMVs could also display relevant antibodies against the PD-1/PD-L1 immune inhibitory axis to block checkpoints and avoid immune escape in cancer^[34,38]^. In addition, the combination application of CMVs, possessing the capability of targeted therapy with immune checkpoint inhibitors, also demonstrated significant tumor suppression effects. The combination reagent CD64-NVs-aPD-L1-CP prepared *via* incubating CD64-CMVs with PD-L1 antibody and cyclophosphamide, simultaneously interrupted the immune escape mediated by PD-L1 and regulated the inhibition of regulatory T cells (Tregs), suppressing the tumor growth and prolonging survival time^[64]^. Similarly, IL-15/IL-15R NVs packaging the PD-1/PD-L1 inhibitor 1 simultaneously enhanced the activation and proliferation of tumor-infiltrated T cells while preventing PD-L1-mediated CD8^+^ T cell exhaustion^[65]^. In addition to targeting immune cells or normal cells to overexpress PD-1 on CMVs membrane for maneuvering the antitumor effects, tumor cells can also be targeted for interfering with the expression of genes associated with tumor progression. For instance, EVs loaded with siRNA against PD-L1 show exceptional capability of recruiting tumor-associated myeloid cells, increasing CD8^+^ cytotoxic T cells activity, and suppressing tumor growth with the existence of RGD, which significantly enhanced the targeting efficiency to murine GBM^[66]^.

#### CMVs in Chemotherapy

Chemotherapy, being one of the most pivotal strategies for cancer clinical treatment, has shown substantial efficacy in impeding tumor progression. However, decades of clinical data have proved that although chemotherapeutic agents effectively eliminate tumor cells and promote tumor regression, they inflict damage to normal cells within the body and lead to adverse effects including cytokine release syndrome, organ damage, and other cancer tumor lysis syndromes in patients, making the prognosis of patients after chemotherapy worse^[67]^.The therapy even caused tumor spontaneous metastasis in certain types of cancer models^[68-71]^. In recent years, synthetic drug delivery carriers such as liposomes have enhanced the efficacy of chemotherapy drugs and alleviated their inherent toxicity^[72]^. Nevertheless, the potential immunogenicity, restricted release efficiency, and undesired side toxicity of liposomes cannot be ignored. Genetically engineered CMVs are expected to provide novel avenues for chemotherapy therapeutic with significantly reduced toxicity and attenuated chemotherapeutic drug resistance. Furthermore, the combination of spatially controllable targeting ability with biocompatibility ensures that genetically engineered CMVs-based drug reagents serve as a robust platform for the delivery of chemotherapy drugs. Genetically engineered EVs derived from imDCs, displaying the LAMP-2B fused with αv integrin-specific iRGD peptide (CRGDKGPDC), were concurrently loaded with DOX. The iRGD-exosomes showed the excellent targeting and DOX convey capacity to αv integrin-positive cancer cells, resulting in the deceleration of tumor growth without distinct toxicity^[40]^. Liu *et al.* developed CL4-modified exosomes in order to target DARS-AS1 siRNA and DOX to triple-negative breast cancer (TNBC) cells^[73]^. The downregulation of DARS-AS1 improved the susceptivity of TNBC cells to DOX, furthering the synergetic antitumor response. The study proved that the transport of DARS-AS1 *via* siRNA EXOs-CL4 holds significant potential as a novel DOX-resistant treatment strategy^[73]^. A study developed exosomes loaded with PTX, which are partially combined with incorporated aminoethylanisamide-polyethylene glycol (AA-PEG) vector to specifically bind to the sigma receptor. The AA-PEG-exoPTX significantly accumulated where the tumor cells have been established and improves the drug’s therapeutic outcomes^[44]^. As mentioned earlier, the cRGD-Exo-PTX was prepared and confirmed to exhibit much better performance in improving the PTX’s pharmaceutical effect *via* enhanced targeting capability in GBM^[42]^.

The synergistic application of chemotherapy with other cancer therapies has sparked fresh insights into the realm of cancer treatment. For example, the bone marrow mesenchymal stem cell (BM-MSC) exosomes charged with galectin-9 siRNA and oxaliplatin (OXA) prodrug, integrated immunotherapy with chemotherapy and attained notable therapeutic efficacy in tumors. The combined carrier provoked potent antitumor responses by increasing the infiltration of antitumoral cytotoxic T lymphocytes and promoting drug accumulation at the tumor site, reversing the immunosuppressive TME^[74]^. In addition to the aforementioned, ultrasonication was utilized to co-encapsulate photosensitizer (ICG) and DOX into genetically engineered CMVs. The drug-loaded CMVs exhibit significantly improved antitumor therapeutic outcomes^[33]^.

#### CMVs for Radiotherapy

Technological advancements over the past decades have elevated radiotherapy to become one of the most technologically intense disciplines in medicine^[75]^. Benefiting from this, the toxicity and adverse effects of radiation therapy have been minimized. Nevertheless, the issue of resistance following radiotherapy remains unresolved. In response to the challenge of radiation therapy resistance, scientists are exploring innovative strategies, including the application of combination therapies, the development of drugs targeting resistance mechanisms, and the use of novel adjuvant approaches including engineered exosomes. For instance, exosomes loaded with miR-34c demonstrated a strong inhibition in invasion, migration, and proliferation of nasopharyngeal carcinoma (NPC) cells. Furthermore, these engineered exosomes significantly heightened radiation-induced apoptosis in NPC cells, suppressing tumor progression and enhancing the efficacy of radiotherapy^[76]^.Similarly, the overexpression of miR-197-3p by exosomes resulted in a discernible reduction in proliferation and migration capabilities of nasopharyngeal carcinoma (NPC) cells *in vitro*, along with decreased tumor growth and radioresistance *in vivo*. The authors substantiated that EXO-miR-197-3p effectively impeded the progression and radioresistance activation of AKT/mTOR phosphorylation and orchestrating autophagy *via* HSPA5 mediation^[77]^.

#### CMVs for Gene therapy

Recently, considerably novel strategies for the clinical application of engineered-CMVs-based gene therapy have increasingly intrigued researchers. Genetically engineered CMVs facilitate gene therapy targeting the diseased genome with high specificity and admirable flexibility. The gene-modulating strategies primarily include improved viral vectors and therapeutic RNAs. Numerous studies have been dedicated to investigating the functions of RNAs delivered by genetically engineered CMVs. As claimed by previous results, RNAs loaded by genetically engineered CMVs indeed possessed the normal biofunction, effectively modulating expression of target genes associated with tumor development and establishment^[51-53]^. The specific siRNA was delivered by EVs to oncogenic *KRAS*^G12D^ , and the CD47 on exosomes prevented siRNA from phagocytosis by monocytes and extended the half-life of exosomes in the circulation. Thus, their combined utilization significantly inhibited the progression of pancreatic cancer^[78]^. Furthermore, genetically engineered CMVs-based gene therapy combined with other therapeutic strategies could trigger a more intensive antitumor immune response and improve the prognosis^[46]^.

### Genetically engineered CMVs for Autoimmune Diseases

The attacks launched by the immune system on normal components of the body cause autoimmune diseases^[79]^. In the past decades, autoimmune diseases have emerged as the third-largest category of chronic disease, profoundly impacting the quality of life of patients^[80]^. Therapies derived from CMVs have also brought new light to advancing the treatment of autoimmune diseases.

#### Type 1 diabetes mellitus (T1DM) treatment

Type 1 diabetes is a chronic disease caused by the autoimmune destruction of β cells responsible for insulin production in pancreas, which is mediated by autoreactive CD4^+^ and CD8^+^ T cells infiltrating the islets^[81,82]^. Inhibiting the autoreactive T cells to protect β-cells from damage is a promising strategy for T1D therapy. The PD-1/PD-L1 axis, widely acknowledged as the immune checkpoint signal, inhibits the activity, induces the exhaustion of T cells, and is promising to autoimmune attack in T1D. For example, New-Onset Type 1 Diabetes could be reversed by engineered immunosuppressive platelets expressing PD-L1. The engineered platelets not only may inhibit the effects of pancreatic aggressive T cells, but also increase the proportion of Tregs maintaining immune tolerance^[83]^. Becker *et al.* designed EVs to regulate T cell effects in the model of T1D. The K562, a lymphoblast cell line, was genetically engineered to express HLA-A*02 (HLA-A2) along with costimulatory CD80 and/or coinhibitory PD-L1^[84]^. EVs packaging PD-L1 caused an immunosuppressive response, which restrained activation and cytotoxicity of CD8^+^ T cells^[84]^.

The favorable therapeutic outcome of EVs on autoimmune diseases has attracted widespread attention and may provide a novel prospective approach for T1D treatment. The exosomes secreted by adipose tissue macrophages carrying miRNAs lead to glucose intolerance and insulin resistance^[85]^. The adipose tissue discharged exosomes and exosomes were absorbed by mononuclear cells, leading to their differentiation into active macrophages with increased release of pro-inflammatory cytokines such as TNF-α. The activation intermediated by exosomes involves the TLR4/TRIF pathway and contributes to insulin resistance^[86]^. Additionally, exosomes secreted by M2 polarized macrophages from bone marrow contained miRNA that contributes to improved glucose tolerance and insulin sensitivity. Ying *et al.* found that miR-690 is highly expressed in M2 BMDM and has an insulin-sensitizing function. It suggested that miR-690 could be a novel insulin sensitizer in metabolic disease^[87]^. Conversely, exosomes produced by adipose-derived stem cells promoted insulin sensitivity and reduced inflammation by inducing an anti-inflammatory M2 phenotype. This confirmed that exosomes are pivotal in regulating immunity and maintaining metabolic equilibrium. The miRNAs carried by these exosomes modulated TGF-β and Wnt/β-catenin signaling, which provide the potential treatment sites and are vital in the progression of chronic inflammation^[88]^. The studies have confirmed that CMVs containing miRNAs improved insulin sensitivity and reduced the inflammatory response. These findings revealed the importance of CMVs in mediating the crosstalk between adipose tissue, inflammation, and insulin resistance.

#### Systemic lupus erythematosus treatment

Systemic lupus erythematosus (SLE), characterized by persistent and excessive inflammation and autoantibody production, is an autoimmune disease involving multiple systems and organs^[89]^. Generally, massive autoantibodies against self-antigens produced by overactive B cells will organize immune complexes with nucleic acids and complements, which accumulate in the skin, glomeruli, and other tissues, ultimately leading to the organ damage or dysfunction^[90,91]^. Although live MSCs have shown impressive efficacy in clinical practice as therapy, they pose challenges such as senescence, low cell survival rates, varying degrees of immune rejection, and potential carcinogenicity^[92]^. Due to their cell-free nature, exosomes present more advantages over MSCs while delivering comparable therapeutic effects, making them a new treatment option^[93]^. Hence, genetically engineered exosomes have progressively garnered the attention of researchers. For example, CD40 was presented on the surface of genetically engineered NIH 3T3 cells to harvest CD40-NVs. CD40-NVs disrupted the CD40/CD40L costimulatory signal axis on B cells, thereby inhibiting their capability to produce antibodies. Concurrently, it limited the normal formation of germinal center structure. Furthermore, this work encapsulated the immunosuppressive drug mycophenolate mofetil (MMF) into aEVs, and it indicated that the combination regent could be employed to deplete immunocytes^[94]^. Similarly, it is noteworthy that Xu *et al.* developed the PD-L1^+^ MSC-derived EVs (MSC-EVs-PD-L1) with immunosuppressive properties, reprogramming the local immune microenvironment proximity to an infected organism^[95]^. It has been confirmed that the above engineered EVs have the ability to reconstitute the inflammatory microenvironment and show promising therapeutic effects in the disease models of UC and psoriasis in mice^[95]^. It is believed that the application of this similar idea for the treatment of SLE will be a promising strategy.

### Genetically engineered CMVs for complications of type 2 diabetes mellitus therapy

#### Chronic diabetic wounds due to diabetes

Type 2 diabetes mellitus (T2DM) is distinguished by hyperglycemia resulting from impaired insulin secretion and insulin resistance^[96]^. Chronic diabetic wounds are one of the most recognized challenging chronic diabetic complications in clinical diagnosis and therapy worldwide. The wound of diabetic patients is particularly difficult to heal, resulting in disability and high mortality, which significantly impacts the physical and mental health and treatment prognosis of patients^[97-99]^. The pathogenesis of this type of wound is complex, and the main feature is that continuous hyperglycemia leads to excessive oxidative stress in tissues, inhibits angiogenesis, and prolongs inflammatory response around the wound^[100]^. However, there is still a lack of forceful treatment within the clinical domain. Recently, more and more evidences has been reported that the delivery of engineered exosomes containing biotherapeutics is a prospective strategy for diabetic wound healing. In particular, the positive therapeutic effects of EVs secreted by MSCs (MSC-EVs) on autoimmune diseases have attracted considerable attention. This may provide a promising new strategy for T1D treatment, and the healing of chronic diabetic wounds in T2DM is no exception. MSC EVs loaded with the miR-155 inhibitor promoted keratinocyte migration and showed synergistic effects on anti-inflammatory meanwhile. Moreover, negative regulation of miR-155 enhanced collagen deposition, angiogenesis, and re-epithelialization, leading to accelerated wound healing^[101]^. MSCs, as original cells, are engineered *via* genetic modification to create MSC-EVs overexpressing the long non-coding RNA HOX transcript antisense RNA (HOTAIR). The results demonstrated that exosomes secreted with increased HOTAIR contributed to angiogenesis and wound healing in chronic wounds^[102]^.

A recent study published by Chu *et. al* built miR-17-5p-overexpressing MSCs and identified the therapeutic effect of miR-17-5p on chronic diabetic wound healing. The overexpressed miR-17-5p caused Hypo-sEVs to possess the functions of targeting, inhibiting ROS/MAPK activity, and blocking neutrophil extracellular traps (NET) formation which has been increasingly recognized as an extremely unfavorable factor in diabetic wound healing. MiR-17-5p overexpression is conducive to recovering the fibroblasts’ function and alleviating ER stress mediated by NET formation, and is recognized as a promising NET-targeting treatment based on MSC-EVs in the treatment of chronic diabetic wounds^[103]^.

#### Fracture risk due to diabetes

Diabetic bone marrow-derived macrophages (dBMDM), transfected with miR-144-5p inhibitor, significantly downregulated the expressive abundance of miR-144-5p in exosomes so that it rescued the adverse impact of dBMDM-exos on bone repair and regeneration. The results presented that miR-144-5p loaded into dBMDM-derived exosomes restrained osteogenesis differentiation and negatively impacted fracture healing^[104]^. Researchers upregulated the abundance of miR-140-3p in BMSC-Exos through genetic engineering and showed that compared with diabetes mellitus-Exos, miR-140-3p-Exos promoted the osteoblast genesis function of BMSCs by inhibiting the expression of plexin B1, thereby accelerating the diabetic bone wound healing and promoting bone regeneration^[105]^. Moreover, given the significance of improving the immunosuppressive microenvironment for fracture repair, strategies employing CMVs for the suppression of overactive immune cells can also be exploited^[106]^. For example, exosomes with enriched concentrations of PD-L1 generated by genetically modified umbilical vein endothelial cells were delivered and released to the surrounding microenvironment *via* injectable hydrogel. It was demonstrated that the PD-L1-enriched exosomes specifically inhibited the activity of T cells in peripheral lymphatic tissues without affecting the activation of T cells in distant immune organs. Meanwhile, it promoted MSCs’ differentiation towards osteogenesis in the presence of T cells, and promoted adequate fracture healing^[107]^.

### Genetically engineered CMVs for Cardiovascular diseases

Cardiovascular diseases remain the most prevalent cause of death and chronic disability worldwide^[108]^. Cell therapy has been recognized as the most potential treatment for cardiomyocyte injury. However, the mechanism underlying stem cell therapy for damaged hearts is the acute inflammatory wound healing response rejuvenating necrotic areas of the heart, which is not associated with the production of new cardiomyocytes, presumed to work *via* paracrine mechanisms^[109,110]^. It is worth emphasizing that exosomes executing unique biological functions from original cells hold potential applications im cardiovascular treatment^[111]^. Currently, there are more extensive reviews available regarding the functions and application of CMVs in the context of cardiovascular diseases^[112-114]^.Genetic engineering can be employed to cargo mRNA and other types of nucleic acids into cells, where nucleic acids including miRNA or small interfering RNA, can be subsequently packaged into exosomes. Overexpressing miRNA-133 promotes the therapeutic efficacy of mesenchymal stem cells for acute myocardial infarction^[115]^. The miRNA-181a carried by MSC-exo exercised an influence over immune-suppressing regulation, and when combined with the targeting capability of MSC-exo, it exerted a more valid treatment effectiveness on myocardium ischemia/reperfusion (I/R) injury. Similarly, EVs enriched with miRNA-181a promoted Treg polarization of peripheral blood mononuclear cells and increased the EF of infarcted mouse hearts by 12%^[116]^. CD47-EVs collected from MSCs were generated by loading purified CD47-EV with miR-21a. Following intravenous administration of miR-21a-loaded CD47-EVs, their accumulation in the heart was observed. These vesicles effectively attenuated myocardial I/R apoptosis and alleviate cardiac inflammation, ultimately contributing to the restoration of cardiac function post-I/R injury^[117]^.

## CONCLUSION

In summary, we retrospect the preparation approaches, the modified strategies for genetically engineered CMVs, and their potential applications as a novel drug delivery system in treating cancer, diabetes, and other diseases. In comparison, unmodified CMVs retain certain attributes of original cells, with research primarily focused on exploring their basic biological characteristics^[4,7]^. Additionally, due to the presence of specific biomarkers, unmodified CMVs play a crucial role in clinical diagnosis^[118]^. However, unmodified CMVs lack specificity and exhibit poor targeting capacity. Engineered exosomes show promising clinical potential across diverse fields, encompassing tumors, diabetes, and cardiovascular diseases. They have demonstrated enhanced therapeutic effects and improved targeting capabilities compared to their natural counterparts^[119,120]^. In contrast to other engineered methods of original cell modification, genetic engineering has the advantage of serving as carriers for protein and nucleic acid therapeutics simultaneously^[121,36]^. Moreover, the engineered CMVs with receptors or ligands overexpressed on their surface can execute cell signal transduction functions, enabling them to regulate the cell function, activity, and even proliferation of the target receiver cells. Thus, some engineered CMVs with immune checkpoint ligands or receptors could modulate the immunocytes such as dendritic cells and effector T cells, making these CMVs efficacious biotherapeutics for treating cancer and autoimmune diseases. However, genetically modified CMVs have some limitations, such as inefficient packing and damage to the activity of donor cells. Additionally, the types of original cells that can be modified are limited. Moreover, it is essential to acknowledge that the research field of genetically engineered CMVs’ clinical application is still in the early stages without too many strategies reaching clinical transfection. The exoASO-STAT6 (CDK-004), exolL-12^TM^(CDK-003), and exoSTING (CDK-002) from Codiak BioSciences has entered the phase 1 clinical trial, and the clinical trial data is of great concern^[122,123]^.

All candidate preparation methods introduce heterogeneity and result in component loss in CMVs. Thus, it must be noted that direct comparative studies about the methodology are necessary to support current conclusions. In addition, safety in clinical applications should also be a key concern in future studies. It is imperative to conduct comprehensive testing to assess potential issues, including the potential loss or alteration of the original exosome contents and the inadvertent introduction of unwanted substances. Moreover, the quantity of genetically engineered CMVs that can be obtained is limited, making large-scale production cost-prohibitive. It recommends improving cell culture conditions during the large-scale production of genetically engineered CMVs to enhance yield. Additionally, genetically engineered CMVs exhibit immunogenicity, and the current limitations associated with cell line-derived membrane vesicles constrain their application in humans. Genetically engineered CMVs, expressing specific proteins, are susceptible to clearance by the liver *in vivo*, also restricting their targeting capabilities for therapeutic purposes. Hence, choosing low-immunogenicity cells such as MSCs as donor cells or utilizing genetic engineering methods to knock out immunogenic factors aims to unlock the therapeutic potential for the application of genetically engineered CMVs in human disease therapy. While facing challenges such as technical hurdles in achieving optimal preparation efficiency, the necessity for clinical validation, ongoing research, and innovation offers substantial promise for the advancement of exosome-based drug delivery systems. These engineered systems have the potential to serve as efficacious strategies for enhancing disease treatment.

## DECLARATIONS

### Copyright

© The Author(s) 2024.
